# A Case in Practice Supposed to Be an Abscess of the Brain

**Published:** 1887-07

**Authors:** Howard J. Williams

**Affiliations:** Macon, Ga.


### A CASE IN PRACTICE SUPPOSED TO BE AN ABSCESS OF THE BRAIN.*

BY HOWARD J. WILLIAMS, A. M., M. D., MACON, GA.

The following interesting case came under my observation recently:

W. D., white, aged 40 years, locomotive engineer; family history good; habits rather intemperate; no history of syphilis; residence in an extremely malarial locality. About the first of October, 1886, he came to Macon to the house of some friends and stated that he was sick and suffering with severe headache. He said that he had sometime previously been hurt in the head while working under his engine. He was stunned for a few moments, but got up and went on with his work. Whether there was at that time any bleeding from the nose he did not state. Since then, however, he has been troubled with pain in his head and a disposition to vertigo. He seemed stupid, yet answered questions intelligently, but slowly, as though it required an effort to concentrate his thoughts. He had fever, was nauseated, and at times vomited, tongue coated, appetite seemed good, though he appeared somewhat reduced in flesh and strength.

Not improving under domestic treatment, a physician was called, who pronounced it a case of malarial poisoning and prescribed the ordinary remedies for malaria. As he did not return to see the patient, I was called in on the 23d day of October. He was then suffering with intense frontal headache, vertigo and dullness of mind, with at times low muttering delirium and

*Read before the Macon Medical Society, January 4th, 1887.

a disposition to resist any interference. There was only a slight rise of temperature, 99.8°; pulse slow and full, 60 to 64; breathing slow and laborious, 12 to 14 respirations; skin dry and harsh, tongue coated, nausea and occasional vomiting; appetite moderately good when food was given him, but he never expressed any desire for food; pupils contracted, and there was some photophobia. There was no paralysis, yet there was an inability to control either bladder or bowels. There was neither anæsthesia nor hyperæsthesia.

The temperature, after a few days, gradually declined to 98.6° without the use of quinine or anti-periodics. (My treatment was anodyne and expectant). The pain in the head increased, and though constantly present, paroxysms would occur when the patient would be in the deepest agony. During these paroxysms there would be no acceleration of pulse or respiration nor increase of temperature. The dullness of mind deepened and passed into coma. The only expressions that could be obtained from him were those of pain. Periods of excitement would occur, when the patient would get out of bed and attempt to walk about the room, but could not maintain himself. On two occasions, however, he succeeded in getting out of the room, and even went out into the yard, but had to be carried in.

About 6 a. m. on November 2d, he had, without an effort or premonition, a sudden discharge of very offensive greenish pus from his nose. Following this discharge, the breathing became stertorous and the pulse irregular. The left eye became dilated, while the right was contracted, and both insensible to sight. The right side of the face and the right extremities were paralyzed. The temperature rose in a few hours from 98.6° to 103°. There were no convulsions, and death occurred six hours after the discharge of pus.

What was the cause of the above train of symptoms?

It could not be malarial fever, for there was no evidence of periodicity in the symptoms, and the usual treatment for malaria, quinine, failed to give relief. Nor could it be typhoid fever, since there were no intestinal symptoms, and the temperature record did not present the daily variations of that disease. That there was no disease of kidneys, liver, spleen, heart or lungs to give rise to the above nervous symptoms was evident, since nothing abnormal could be found by careful examination. Evidently the symptoms indicated disease located in the cavity of the skull, some local irritation or pressure on the brain. That it was not a chronic disease, tumor or new growth seemed evident, since the history was of short duration and the symptoms of comparatively rapid onset. The fever, delirium and rapid wasting indicate an acute disease. Intense headache and vertigo, the pain being located in the anterior portion of the head, the disordered intellect and disposition to resist interference, the slow respiration and pulse; without disorder of the special senses, paralysis of motion and sensation or convulsions would point to pressure on the anterior lobes.

An abscess of the anterior portion of the brain would give rise to the above symptoms, and I was very much of the opinion that such was the case, and that it resulted from the blow or injury which he received while at work. It is true that there was no fracture of the bones or injury to the soft parts following the blow, nor was he compelled to stop work, and an interval elapsed before the final illness occurred; yet the history of the case, first an injury, followed by constant headache and vertigo, with a period of fever with nervous symptoms and gradually deepening coma, then by a suddenly developed paralysis, attended with a discharge of greenish pus from the nose, and finally a termination in death, would suggest an abscess rupturing into the lateral ventricles, and at the same time rupturing into the nose through the ethmoidal bone. I will state, parenthetically, that there was no chronic disease of the bones of the nose during the patient's life to give rise to the pus, nor any middle-ear disease.

Cerebral inflammation, ending in suppuration and abscess, may follow a concussion or contusion of the brain and its membranes, even though the bones are not fractured and the external soft parts receive no injury, and inflammation and abscess following external violence is often latent, weeks, even months, intervening before symptoms of the disease develop. Often, too, in concussion, the seat of contusion is at a point on the brain opposite to the seat of external violence, a result of *contre-coup* or forcible import of the brain on the inner surface of the skull, which force is sufficient often to fracture the bone. In the present case the blow was on the forehead, while the abscess was on the under-surface of the anterior lobes of the brain, and the force was perhaps sufficient to fracture the ethmoid, giving an exit for the escape of the contents of the abscess cavity.

In conclusion, I suspected abscess of the brain before the discharge of pus and paralysis, but the occurrence of these together confirmed my suspicions. I regret that a *post mortem* examination could not be had, as I think my diagnosis would have been confirmed.

